# Fingolimod reduces circulating tight-junction protein levels and in vitro peripheral blood mononuclear cells migration in multiple sclerosis patients

**DOI:** 10.1038/s41598-018-33672-9

**Published:** 2018-10-18

**Authors:** Pasquale Annunziata, Chiara Cioni, Gianni Masi, Maristella Tassi, Giuseppe Marotta, Sauro Severi

**Affiliations:** 1 Department of Medicine, Surgery and Neurosciences, University of Siena, Siena, Italy; 2 Stem Cell Transplant and Cellular Therapy Unit, University Hospital, Siena, Italy; 3 Neurology Unit, San Donato Hospital, Arezzo, Italy

## Abstract

There are no data on the effects of fingolimod, an immunomodulatory drug used in treatment of multiple sclerosis (MS), on circulating tight-junction (TJ) protein levels as well as on peripheral blood mononuclear cells (PBMC) migration. Serum TJ protein [occludin (OCLN), claudin-5 (CLN-5) and zonula occludens-1 (ZO-1)] levels, sphingosine-1 phosphate 1 (S1P_1_) receptor expression on circulating leukocyte populations as well as *in vitro* PBMC migration were longitudinally assessed in 20 MS patients under 12-months fingolimod treatment and correlated with clinical and magnetic resonance imaging (MRI) parameters. After 12 months of treatment, a significant reduction of mean relapse rate as well as number of active lesions at MRI was found. TJ protein levels significantly decreased and were associated with reduction of S1P_1_ expression as well as of PBMC *in vitro* migratory activity. A significant correlation of CLN-5/OCLN ratio with new T_2_ MRI lesions and a significant inverse correlation of CLN-5/ZO-1 ratio with disability scores were found. These findings support possible *in vivo* effects of fingolimod on the blood-brain barrier (BBB) functional activity as well as on peripheral cell trafficking that could result in avoiding passage of circulating autoreactive cells into brain parenchyma. Circulating TJ protein levels and respective ratios could be further studied as a novel candidate biomarker of BBB functional status to be monitored in course of fingolimod as well as of other immunomodulatory treatments in MS.

## Introduction

In multiple sclerosis (MS), the expansion of activated and autoreactive lymphocyte clones adhering to blood-brain barrier (BBB) endothelium and subsequently migrating into the brain parenchyma is thought to be a fundamental step of cascade of events leading to inflammatory damage to myelin and axons^1^. Interendothelial tight junction (TJ) proteins such as occludin (OCLN), claudin-5 (CLN-5) as well as zonula occludens-1 (ZO-1), contribute to maintenance of the blood-brain barrier (BBB) anatomical and functional integrity^2^ and are subjected to shedding and release becoming detectable in biological fluids such as serum and thus representing a possible *in vivo* marker of BBB functional integrity as recently found in ischemic stroke^3^. Fingolimod (FTY720) phosphate, approved for treatment of highly active relapsing-remitting (RR) MS, acts as a functional antagonist of the sphingosine 1-phosphate (S1P) receptor subtypes and its binding to S1P_1,_ mainly expressed on the lymphocyte surface, results in inhibition of lymphocyte leakage from lymph nodes thus determining a reversible redistribution of pathogenic lymphocytes as well as reducing their recirculation into the central nervous system (CNS)^4^. Moreover, in rat experimental autoimmune encephalomyelitis (EAE), experimental evidence of additional effects of FTY720 on BBB repair through reduction of adhesion molecule expression as well as downregulation of other endothelial barrier gene transcripts (ICAM-1, MMP-9, P-selectin, TIMP-1, VCAM-1) has been achieved^5^. Furthermore, pretreatment of immortalized human brain endothelial cultures with FTY720 prevented BBB alterations induced by MS sera^6^. Conversely, targeting endothelial S1P_1_ with FTY720 in a mouse model caused changes in subcellular location of TJ proteins and led to transient BBB opening^7^. However, to date, there is lack of data on the possible effects of FTY720 on serum TJ protein levels in MS patients as potential *in vivo* surrogate marker of BBB repair as well as on peripheral blood mononuclear cells (PBMC) migration. To address these issues, we longitudinally evaluated TJ protein levels in serum of MS patients under FTY720 treatment, measured the S1P_1_ receptor expression on circulating leukocyte and lymphocyte populations as well as assessed *in vitro* PBMC migration. We also correlated these findings with clinical and magnetic resonance imaging (MRI) parameters.

## Results

### Clinical and MRI features

Clinical and demographic characteristics of the patients and normal healthy subjects (NHS) as well as previous disease modifying therapies (DMT) are summarized in Table 1. After 12-months treatment, mean relapse rate (RR) of the patient cohort in the last year prior to treatment significantly decreased by 96% (p < 0.0001). Out of 20 patients, one had a clinical relapse at 9 months of therapy. There was a slight but not significant reduction of disability score by 11% (p = 0.17) (Table 2). No evidence of disease activity (NEDA) at12 months of treatment, as defined in methods, was observed in 13 of 20 (65%) patients. At the end of follow-up, there was a not significant reduction by 53% of mean new T2 weighted lesions (p = 0.34) at MRI. Conversely, a significant reduction by 80% of T1 gadolinium enhancing (GAD^+^) lesions was observed (p = 0.001) (Table 2).

**Table 1:** Baseline clinical and demographic characteristics of the patients and normal healthy subjects.

	Patients	NHS
	(n = 20)	(n = 25)
Gender (F/M)	14/6	13/12
Age (years)	41.9 ± 9	38 ± 6
Disease duration (years)	14.5 ± 9	n.a.
ARR	0.41 ± 0.3	n.a.
EDSS	2.4 ± 1.6	n.a.
**Previous DMT (n)**		
IFN beta	16	n.a.
Glatiramer acetate	4	n.a.

The data are shown as mean ± SD.

NHS = normal healthy subjects; F = female; M = male; ARR = annualized relapse rate; EDSS = Expanded disability status scale; DMT = disease modifying therapy; n = number of subjects; n.a. = not applicable.

**Table 2:** Clinical and MRI features.

	Baseline	T_12_	P
RR	1.2 ± 0.4	0.05 ± 0.2	<0.0001
EDSS	2.4 ± 1.6	2.1 ± 1.2	0.17
New T2 lesions	0.95 ± 1.8	0.45 ± 0.9	0.34
GAD^+^	1.25 ± 2.0	0.25 ± 0.9	0.001

The results are shown as mean ± SD. RR = mean relapse rate; baseline = at the last year prior to fingolimod treatment; T_12_ = at 12 months of treatment; EDSS = expanded disability status scale; GAD^+^  = T1 gadolinium enhancing lesions. For analysis, Wilcoxon matched-pairs signed rank test was used.

### Serum TJ proteins levels

**Figure 1:**
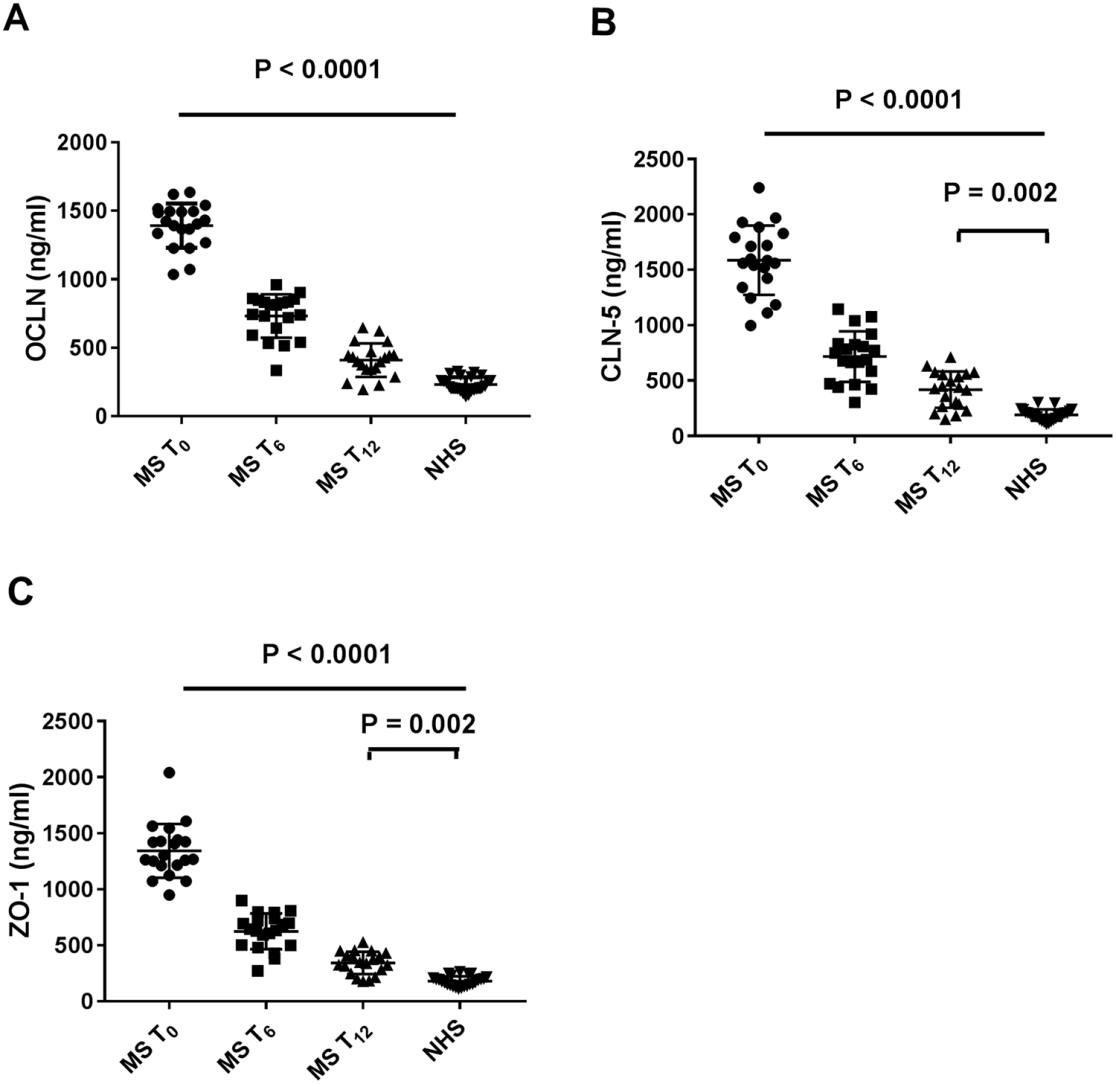
Serum tight junction protein levels in multiple sclerosis (MS) patients (n = 20) during treatment with fingolimod and in normal healthy subjects (NHS) (n = 25): occludin (OCLN) (**A**), claudin-5 (CLN-5) (**B**), zonula occludens (ZO-1) (**C**). T_0_ = baseline time prior to treatment; T_6_ = at 6 months of treatment; T_12_ = at 12 months of treatment. The results are shown as scatter plots with mean ± SD. ANOVA and Tukey post test were used.

**Figure 2:**
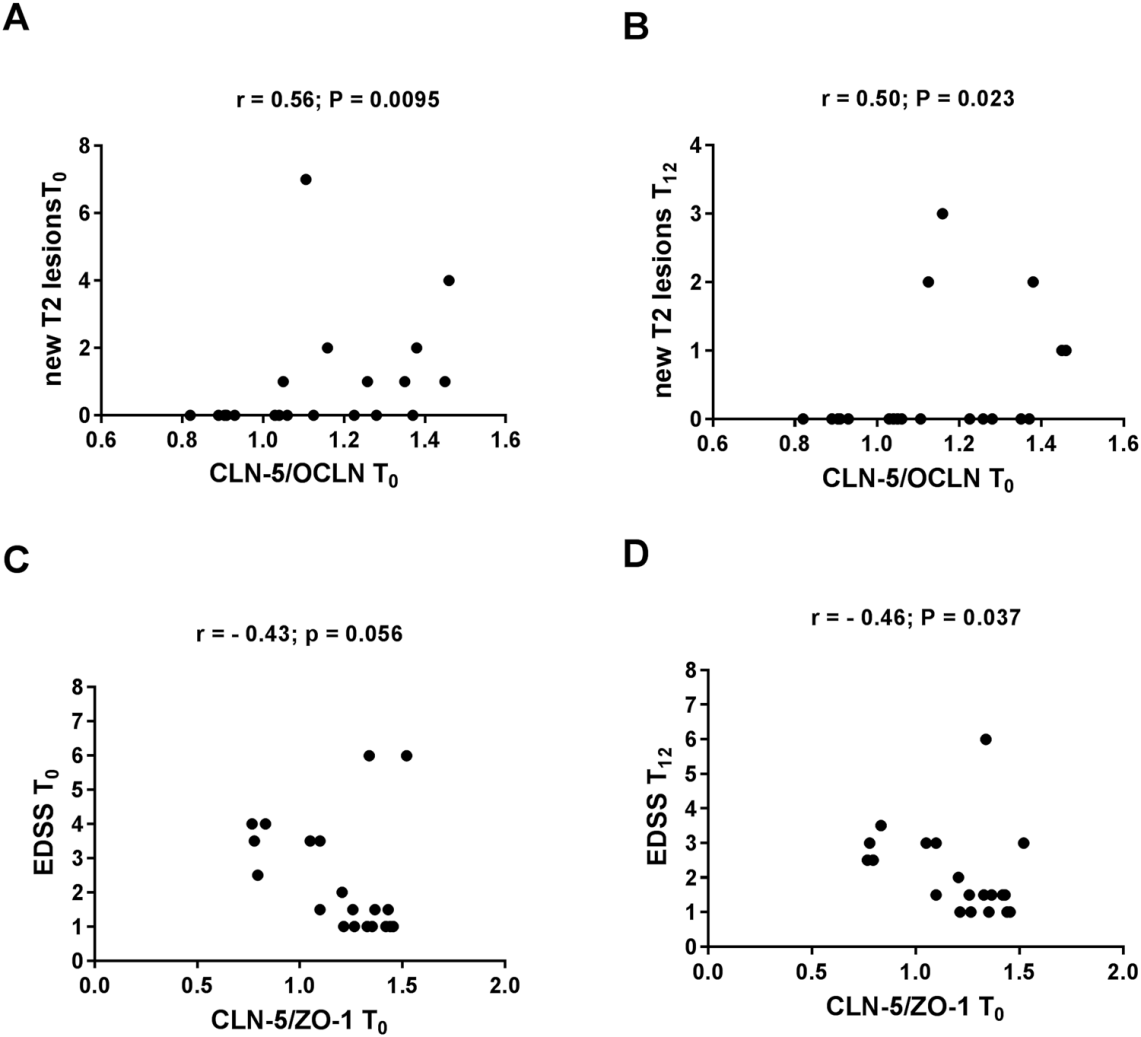
Correlations of serum tight junction protein ratios with clinical and MRI parameters. Correlation of CLN-5/OCLN ratio with new T_2_ weighted lesions at MRI at T_0_ (**A**) and at T_12_ **(B**). Correlation of CLN-5/ZO-1 ratio with EDSS at T_0_ (**C**) and at T_12_ (**D**). T_0_ = baseline time prior to treatment; T_12_ = at 12 months of treatment. CLN-5 = claudin-5; OCLN = occludin; ZO-1 = zonula occludens-1. EDSS = expanded disability status scale. Spearman correlation coefficient was calculated.

All three proteins levels were found to be significantly reduced at 6 months as well as after 12-months treatment. This reduction was substantially similar for all three proteins. In particular, serum OCLN decreased by 47.4% and 70,5% after 6 and 12 months of therapy, respectively (p < 0.0001) (Fig. 1A). Serum CLN-5 significantly decreased by 54.8% and 73.6%, respectively (p < 0.0001) (Fig. 1B) and ZO-1 by 53.5% and 74.5%, respectively (p < 0.0001) (Fig. 1C). However, for all three proteins, the levels at 12 months treatment were significantly higher than those of NHS. There was no significant correlation between single TJ protein levels and clinical parameters such as relapse rate and disability score assessed by EDSS at baseline as well as at 12 months. No significant correlation between single TJ protein levels and neuroimaging parameters at MRI such as new T2 weighted lesions and T1 GAD^+^ lesions was observed. (Supplementary Table S1). However, when calculating CLN-5/OCLN and CLN-5/ZO-1 ratios, we found a significant correlation of CLN-5/OCLN ratio at baseline with the number of new T2 weighted MRI lesions both at baseline (r = 0.56; p = 0.0095) (Fig. 2A) and at 12- months treatment (r = 0.50; p = 0.023) (Fig. 2B). Conversely, there was a high statistical trend towards a negative correlation (r = −0.40; p = 0.056) and a significant inverse correlation (r = −0.46; p = 0.037), of CLN-5/ZO-1 ratio at baseline with EDSS at baseline and at 12- months treatment, respectively (Fig. 2C,D).

### Expression of S1P_1_ in PBMC

**Figure 3:**
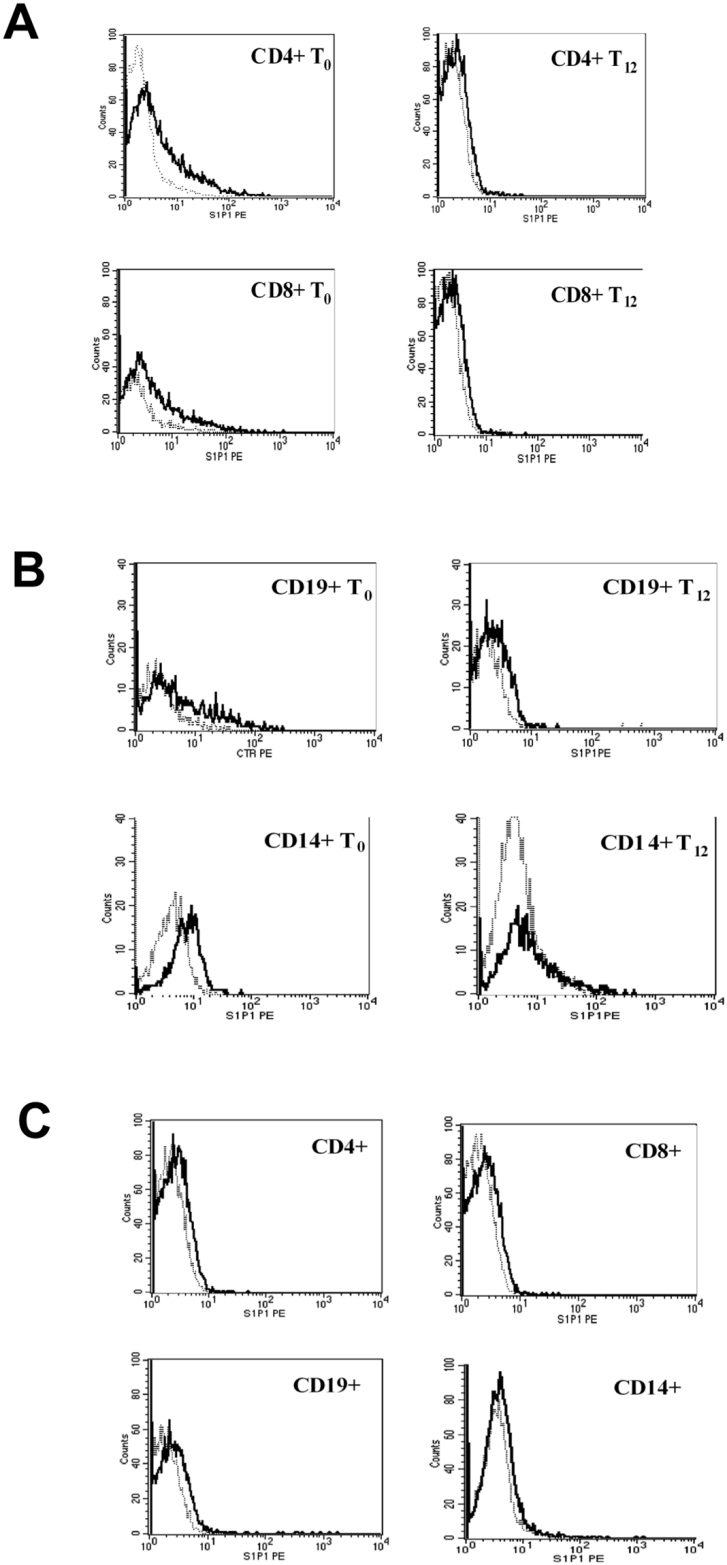
Representative flow cytometry analysis of S1P_1_ receptor in CD4^+^, CD8^+^, CD19^+^ and CD14^+^ cells at baseline (T_0_) and at 12 months of treatment (T_12_) from one MS patient (**A**,**B**) and from one normal healthy subject (NHS) (**C**). Grey dot line: isotype control; black line: specific anti-cell marker monoclonal antibody.

**Figure 4:**
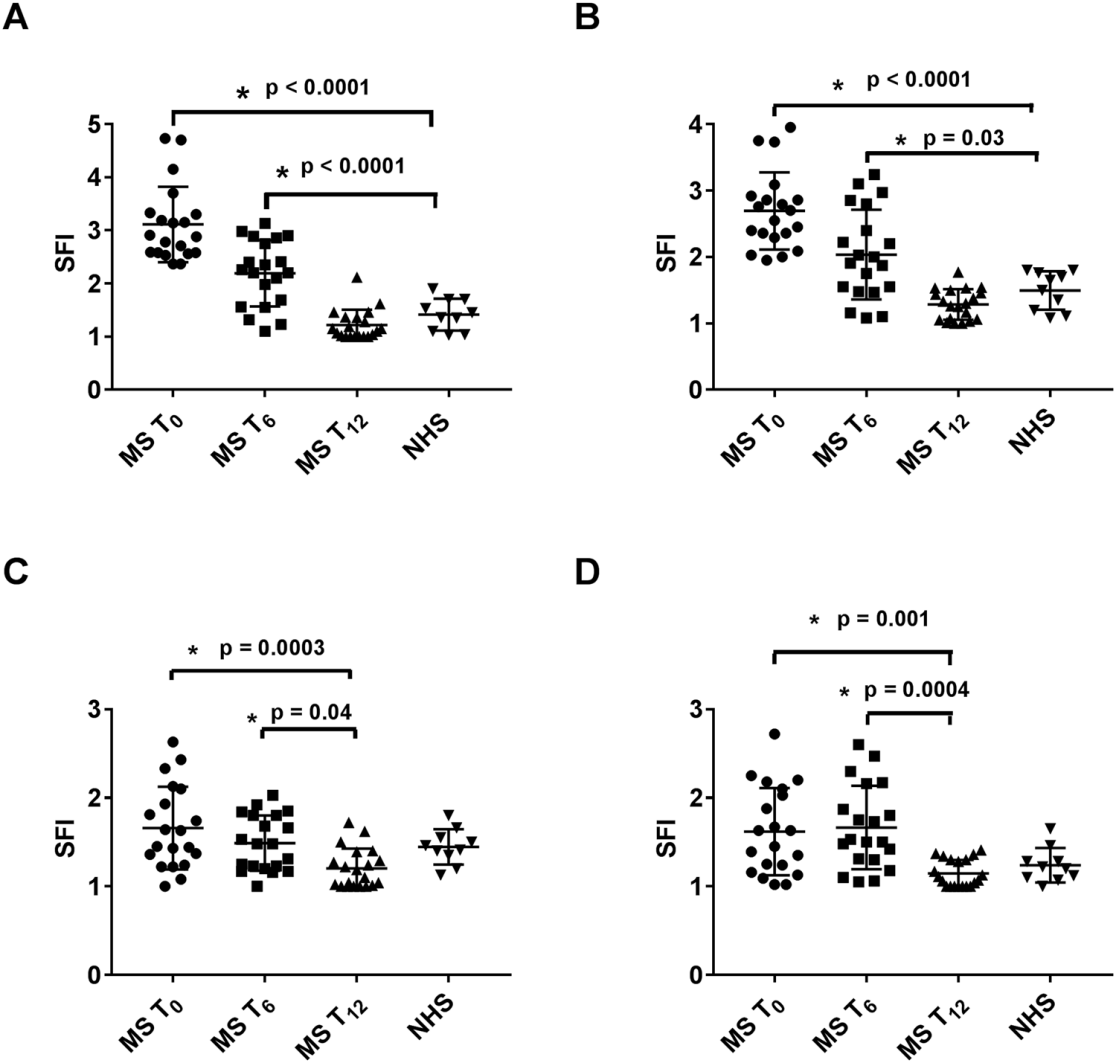
Expression of sphingosine-1 phosphate1 receptor (S1P_1_) in CD4^+^ (**A**), CD8^+^ (**B**), CD19^+^ (**C**) and CD14^+^ cells (**D**) from multiple sclerosis (MS) patients (n = 20) during treatment with fingolimod and from normal healthy subjects (NHS) (n = 10). SFI = specific fluorescence index; T_0_ = baseline time prior to treatment; T_6_ = at 6 months of treatment; T_12_ = at 12 months of treatment. The results are shown as scatter plots with mean ± SD. ANOVA and Tukey post test were used.

Representative flow cytometry analysis of S1P_1_ receptor in leukocyte populations from MS and NHS is shown in Fig. 3. In MS patients, a significantly reduced expression of S1P_1_ by 30% and 61% was observed in CD4^+^ cells at 6 and 12 months, respectively (p = 0.0001) (Fig. 4A). A similar extent of reduction by 24% and 52% was observed in CD8^+^ cells (p = 0.0001) (Fig. 4B). In both subpopulations, S1P_1_ expression extent after 12 months of therapy, reached that of NHS. In CD19^+^ cells, the reduction was 11% and 28%, respectively (p = 0.019) (Fig. 4C) whilst in CD14^+^ cells, the S1P1 expression was unchanged at 6 months but significantly decreased by 29% at 12 months (p = 0.0001) (Fig. 4D). However, in both CD19^+^ and CD14^+^ cells, treatment reduced the receptor expression to values similar to those of NHS.

### Chemotaxis assay

**Figure 5:**
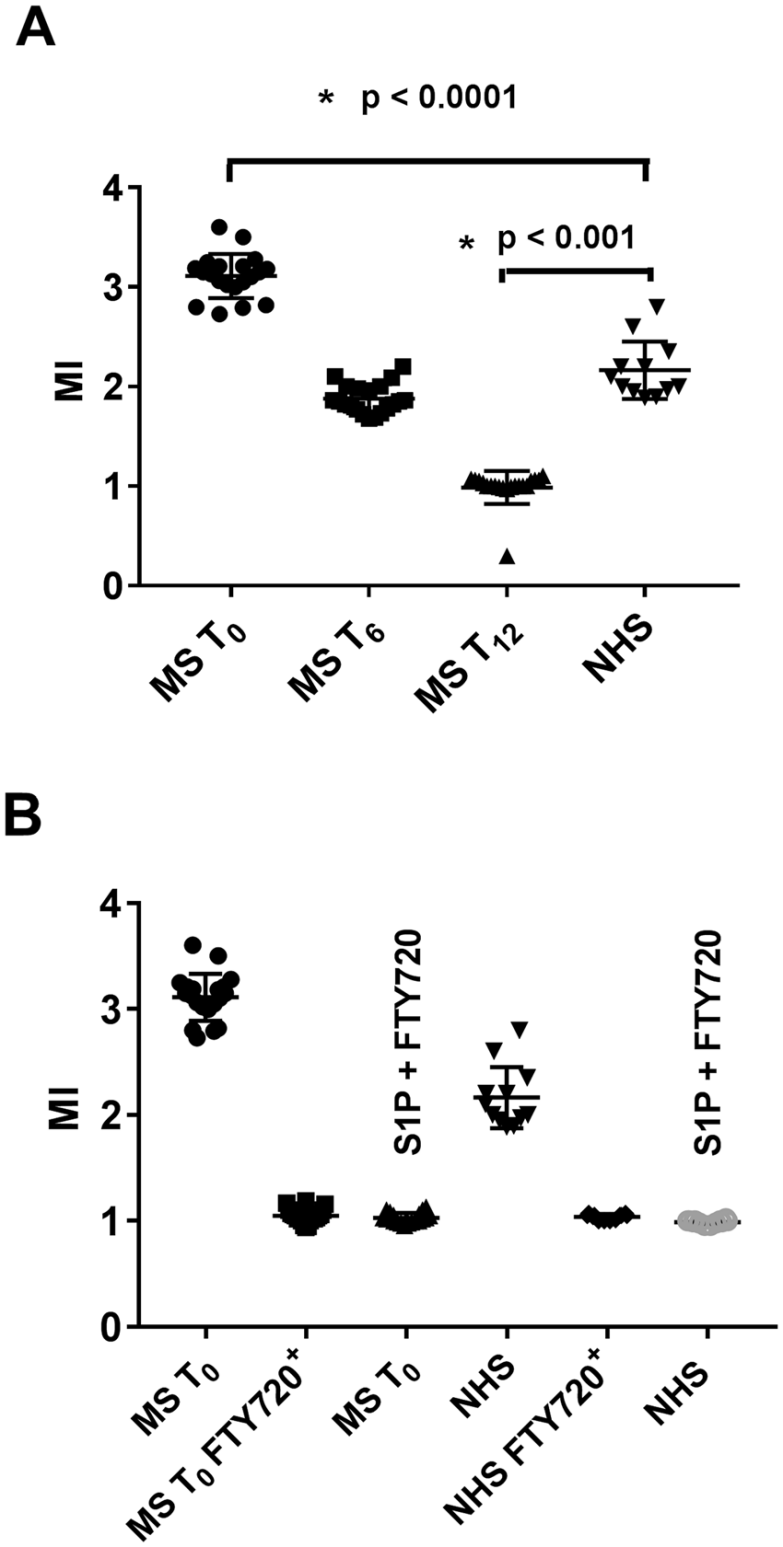
*In vitro* migratory activity of peripheral blood mononuclear cells (PBMC) from multiple sclerosis (MS) patients (n = 20) during treatment with fingolimod and from normal healthy subjects (NHS) (n = 12) (**A**). *In vitro* migratory activity of PBMC from multiple sclerosis (MS) patients prior to start fingolimod treatment and from normal healthy subjects (NHS) prior to and after *in vitro* exposure to fingolimod (**B**). MI = migration index; T_0_ = baseline time prior to treatment; T_6_ = at 6 months of treatment; T_12_ = at 12 months of treatment; S1P = sphingosine 1-phosphate; MS T_0_ FTY720^+^ = PBMC from MS patients at baseline time exposed to fingolimod; NHS T_0_ FTY720^+^ = PBMC from NHS after *in vitro* exposure to fingolimod. S1P+ FTY720 = assays performed with S1P at bottom of the chemotaxis chamber in the presence of FTY720. The results are shown as scatter plots with mean ± SD.

A very significant reduction of PBMC migration rate by 39.5% and 68% was observed at 6 and 12 months, respectively (p < 0.0001) (Fig. 5A). To test whether the effect on migration was specifically due to FTY720 therapy, we assayed PBMCs from NHS (n = 12) or MS patients (n = 20) at baseline after a 24-hour *in vitro* exposure to FTY720 and observed a migration rate similar to that of MS patients at 12 months therapy (Fig. 5B). There was no correlation between the extent of S1P_1_ expression in CD4^+^ (r = 0.03; p = 0.89) and CD8^+^ cells (r = −0.16; p = 0.48) and PBMC migration rate indicating no direct role of lymphocyte S1P_1_ expression on the peripheral cell migration.

## Discussion

In this study, we demonstrated that treatment with fingolimod is associated with significant reduction of circulating TJ proteins levels contributing to the architecture of BBB. BBB changes constitute one of the pathological hallmarks in MS^8^ and are associated with TJ proteins alterations. In MS brains, changes in ZO-1 and OCLN were found in both active lesions and normal-appearing white matter (NAWM)^9^ and ZO-1 was also found altered in inactive chronic lesions^10^. These findings suggest that damage to TJ proteins concurs to cause subtle alterations of the BBB architecture even in areas where the inflammatory process should be exhausted as well as in areas far from active or chronic lesions such as NAWM supporting the concept that BBB function is profoundly compromised in MS and with difficulty normalizes^11^. Moreover, there is experimental evidence that serum from active MS patients reduces the expression of OCLN and VE-cadherin in endothelial cultures suggesting that circulating proinflammatory factors such as cytokines, metalloproteinases as well as reactive oxygen radicals contribute to TJ protein changes and subsequent endothelium alterations^12^. These pathological findings parallel the MS real clinical life where often GAD^+^ lesions at MRI, thought a surrogate marker of BBB alteration^13^, were also detected during clinical stability stages independently of occurrence of new clinical relapses^14,15^. Despite the lack of any correlation between single TJ protein levels and number of GAD^+^ lesions, we, however, detected the highest TJ protein levels at baseline, in patients showing at least one GAD+ lesion or experiencing a clinical relapse at a time close to blood collection and thus being in active disease, consisting with the association of TJ proteins alterations with BBB leakage. However, considering that the average duration of contrast enhancement at a MRI lesion was found to be around 3 weeks^16^ and the different time of blood collection respect to MRI in our cohort, we can’t rule out that TJ protein levels could be lower than those detected and thus may be underpowered to allow to reach significant values at the correlation analysis. Of interest is the significant correlation of CLN-5/OCLN ratio with new T2 weighted lesions at MRI suggesting a common pathway underlying the release of both TJ proteins into the peripheral blood and related to brain inflammatory lesion formation as indicated by detection of new T2 lesions. It has been demonstrated that, at the cerebral microvessels, CLN-5 and OCLN are assembled into oligomers contributing to selective inhibition of the paracellular transport as well as to endothelial cell polarity fundamental for maintaining BBB integrity^17^. Moreover, the significant inverse correlation between CLN-5 /ZO-1 ratio and EDSS suggests that increased release of ZO-1 reduces this ratio and is associated with the complex process underlying the disability accrual. This hypothesis is supported by ZO-1 location at the brain endothelial cell cytoplasm binding both to the occludin-CLN-5 complex and to cytoskeleton proteins^18^. It is likely that the cascade of events causing TJ protein release leads to disassembly of cytoskeleton proteins such as actin thus resulting in BBB breakdown associated with diffuse inflammation and nervous tissue damage and causing MS disability^11,19^.

In our study, the lowest TJ protein levels were detected after 12-months treatment showing a significant decrease of GAD^+^ lesions and thus supporting an *in vivo* effect of fingolimod on BBB functional activity and likely on repair of BBB damage due to neuroinflammation. However, these levels, although reduced, tended to be higher than NHS supporting the persistence of subtle BBB alterations in clinically stable MS patients, even though we can’t rule out that an extension of longitudinal measurement over 12-months treatment could show further level reduction.

Of interest are the findings on the S1P_1_ expression in different leukocyte populations never, to the best of our knowledge, investigated in MS patients. It is demonstrated that reduction of S1P_1_ receptor expression by fingolimod is due to its internalization^20^ and subsequent degradation^21^. In our study, the extent of expression reduction differed between lymphocyte subsets and B cells as well as monocytes, respectively, likely reflecting possible differences in the effects of fingolimod on circulating immunocompetent cells rather than fluctuations of single cell populations counts^22^. Indeed, several distinct modulatory effects on B cell subsets and related cytokine phenotype^23^ as well as on monocytes^24^ were recently found in MS patients under fingolimod treatment. Unique are the findings on PBMC migration suggesting that circulating PBMC not entrapped in lymph nodes by fingolimod, are subjected to changes in the complex pathway of adhesion molecules and cell surface receptors involved in cell adhesion to and passage across BBB endothelium. Experimental evidence of an effect of fingolimod on these molecules has been achieved in EAE where reduction and downregulation of a number of endothelial adhesion molecule transcripts has been shown^5^. In addition, a significant reduction in CXCL13 levels, a chemokine involved in leukocyte cell trafficking, has recently been found in cerebrospinal fluid from MS patients under treatment with fingolimod supporting a modulatory role of this drug in the cell migration^25^. Recently, any insights into the possible underlying mechanisms of fingolimod effects on BBB cell migration have been provided in an animal experimental model by Zhao *et al*. demonstrating that FTY720 blocks *in vivo* leukocyte recruitment into the brain parenchyma by inhibition of brain endothelium activation through reduction of phosphorylation of a number of signaling molecules such as serine/threonine-specific protein kinases, STAT6 and NFkB^26^. In our study, the demonstration of a direct effect of fingolimod on cell migration was provided by the finding that PBMC from NHS as well as MS patients prior to starting treatment, display a significant reduction of migratory activity up to the extent detected in MS patients at 12-months treatment, after *in vitro* exposure to this molecule. Although we were not able to find any correlation between S1P_1_ expression and extent of migration rate, the overlapping of migration index of fingolimod-treated cells with that of untreated cells, suggests however a link of this receptor subtype with migration pathway that remains to be elucidated. A possible limitation of these findings comes from the characteristics of the chemotaxis assay lacking in an endothelial cell monolayer more representative of the BBB architecture *in vivo* and thus not allowing to achieve direct data on BBB transmigration.

In conclusion, our study provides novel data on possible *in vivo* effects of fingolimod on BBB functional activity and repair during neuroinflammation and extends its effect on peripheral blood cell trafficking speculating on a possible reduction of migratory activity of circulating immunocompetent cells across BBB and likely avoiding passage of circulating autoreactive cells into brain parenchyma. Further studies are however needed to confirm this hypothesis. All biomarkers of BBB alteration in MS, investigated to date, resulted poorly sensitive when correlated with the number of GAD^+^ lesions^27^. It is therefore reasonable in searching for new BBB investigating tools, to take into account other quantitative MRI parameters than number of GAD^+^ lesions such as volume of gadolinium enhancement even though different patterns of *in vivo* BBB alteration should be considered in MS^28^. However, circulating TJ proteins levels and respective ratios could be further studied as a novel candidate biomarker of BBB functional status to be monitored in course of fingolimod as well as of other immunomodulatory treatments in MS patients.

## Materials and Methods

### Patients

This is an observational prospective study. Twenty RRMS patients (6 male and 14 female subjects)^29^ were recruited. Twenty-five sex- and age-matched NHS, blood donors at the Blood Transfusion Centre of the Siena University Medical School and giving informed consent, were included as control group. All patients signed informed consent and the study was conducted according to the Declaration of Helsinki and approved by the Ethics Committee of the Medical School of University of Siena. All patients started second-line therapy with FTY720 (0.5 mg/die) according to the criteria approved by Agenzia Italiana del Farmaco (AIFA) including the failure of a first-line therapy or occurrence of highly active MS as demonstrated by at least two clinical relapses in the last year with at least one GAD^+^ lesions or increase in number of T2 weighted lesions at MRI^30^. Number of relapses in the last year prior to enrollment in the study as well as sustained disability score by EDSS^31^ at the enrollment time and after 12 months of treatment were assessed. Relapses were defined as occurrence of new clinical neurological signs or symptoms not associated with fever. Brain MRI at 1.5 Tesla with and without gadolinium infusion was performed according to the standard procedures for clinical follow-up at baseline time and after 12 months of treatment and number of new T2 weighted and T1 GAD^+^ lesions was estimated. In addition, NEDA at 12 months defined as a composite consisting of absence of relapses, no sustained EDSS score progression, and no new or enlarging T2 or T1 GAD^+^ lesions at MRI was assessed.

### Blood collection and PBMC separation

PBMC were isolated by standard density gradient centrifugation procedures on Ficoll-Hypaque from buffy coat preparations of NHS obtained on the same day by Transfusion Centre, as well as from MS patients. In MS subjects, blood sample was drawn at baseline time after a wash out of the first-line therapy ranging from three to four weeks and at 6 and 12 months of FTY720 therapy. In NHS, serum sample was obtained by Transfusion Centre on the same day as blood donation.

### ELISA assay for the assessment of serum levels of claudin, occludin and zonula occludens

Serum TJ proteins (CLN-5, OCLN and ZO-1) were measured by sandwich ELISA using commercial monoclonal and polyclonal antibody pairs, as briefly described. After washing with 0.05 Tween-20 in PBS at pH 7.4 and incubating for 2 hours at room temperature with blocking buffer (3% BSA in PBS at pH 7.4) to block unspecific binding sites, microplates, pre-coated with mouse anti human OCLN monoclonal antibody (mAb) (clone E-5, Santa Cruz, Dallas, TX, USA) or anti human CLN-5 mAb (clone A-12, Santa Cruz) or anti-human ZO-1 mAb (clone 61357, Abcam, Cambridge, UK) (all 5 μg/ml in carbonate buffer pH 9.6) overnight at 4 °C, were overlaid with recombinant OCLN (Abnova, Taiwan), CLN-5 (Abnova) e ZO-1 (Genway Biotech, CA, USA) to generate a reference standard curve (ranging between 0,156 and 10 ng/ml) as well as with MS and NHS sera (1:400 in PBS pH 7,4 - 0,5% BSA) for 2 hours at room temperature (Supplementary Fig. S1). Serum dilution was chosen from an ELISA curve previously obtained from 4 human serum samples (Supplementary Fig. S2). After washing, microplates were incubated for 2 h at room temperature with either polyclonal goat anti-ZO-1 antibody (2.5 μg/ml, Santa Cruz) or rabbit anti-CLN-5 (2,5 μg/ml, Santa Cruz), or peroxidase (POD) conjugated mouse anti-OCLN mAb (clone: OC-3F10, 1 μg/ml, Zymed-Thermo Fisher Scientific,Whaltam, MA, USA). After washing, affinity- purified POD conjugated anti-goat and rabbit IgG (1:10000, all from Calbiochem - Novabiochem, San Diego, CA, USA.) were added in microplates exposed to anti-ZO-1 and anti-CLN-5 mAbs as a secondary antibody for 1 h at room temperature. After washing, 0.1% o-phenylendiamine in 0.05 M citrate buffer at pH 4.5–0.002% H_2_O_2_ was added for 10 min at room temperature in dark. The color reaction was stopped with 1 M H_2_SO_4_ and measured with a photometer at 490 nm wavelength. The assay specificity for each TJ protein was tested in a competitive ELISA with recombinant standard proteins and bovine serum albumin as other unrelated protein (Supplementary Fig, S3). Inter-assay coefficient variability (CV) was 2.39%, 3.50% and 3.40% for OCLN, CLN-5 and ZO-1, respectively. Intra-assay CV was 2.60%, 3.99% and 2.48% for OCLN, CLN-5 and ZO-1, respectively.

### Expression of the S1P_1_ receptor in circulating immunocompetent cells by flow cytometry

The differential expression of S1P_1_ receptor was measured with flow cytometry in leukocyte populations (T cells, B cells and monocytes) by using mAbs capable of recognizing specific cell markers (CD4^+^, CD8^+^, CD19^+^ and CD14^+^, respectively) conjugated with different fluorochromes. In particular, 1 × 10^6^ PBMC in PBS pH 7.4 from each patient or NHS, were labelled with allophycocyanin (APC) -anti-human CD4 (clone: OKT4, 0.6 μg/ml), peridinin-chlorophyll-protein-cyanine5.5 (PerCP-Cy5.5) anti-human CD8 (clone: RPA-T8, 2.5 μg/ml), APC-anti-human CD19 (clone: HIB19, 1.25 μg/ml) and PerCP-Cy5.5-anti-human CD14 (clone: 61D3, 5 μg/ml) mAbs, (all from Affymetrix, eBioscience). After incubation for 15′ at room temperature in dark, cells were then fixed with 1% paraformaldehyde in PBS pH 7,4 and after washing, permeabilized with 0,02% saponin-PBS-1% FCS and labelled with phycoerythrin (PE)- anti-human S1P1/EDG-1 mAb (clone: 218713, 10 µl, R&D Systems). As control isotypes, mouse IgG2bk PE-conjugated (clone:A-1, MyBiosource), mouse IgG1k- PerCP-Cy5.5 -conjugated (clone: p3.6.2.8.1) and mouse IgG2bk-APC-conjugated (clone: eBMG2b) (all from Affymetrics, eBioscience) were used. Cells were analyzed on a FACSCalibur flow cytometer using Cell Quest Pro software (BD Biosciences). The extent of expression was assessed as Specific Fluorescence Index (SFI) calculated as ratio of the mean fluorescence values obtained with the specific anti-S1P_1_ as well as anti-CD markers mAbs to the respective isotype control antibody^32^.

### Chemotaxis assay

To test the effects of FTY720 binding to S1P_1_ on migration of circulating immunocompetent cells, a chemotaxis assay was performed. PBMC were labelled with sulfo-NHS-biotin (Pierce, Rockford, IL) (0.2 mg/ml) for 30 minutes at room temperature according to a previously established method^33^. Biotin-labelled cells (25 × 10^3^) in original culture medium (DMEM-10% FCS) were seeded in the upper compartment of a commercial chemotaxis chamber with 3 μm pore size membranes (NeuroProbe, MD, USA). As chemotactic agent, 100 nM sphingosine 1-phosphate (S1P) (Sigma) alone or, in some assays, in the presence of 100 nM FTY720 (Sigma), was placed in lower chamber. For each sample, as negative control, S1P was omitted in lower chamber. After 3 h at 37 °C, streptavidin-POD conjugate (1:5000) for 1 h at room temperature was added in lower chamber and after washes with PBS pH 7,4 and addition of the substrate 0,1% o-phenylendiamine (OPD) in citrate buffer pH 4,5, the product reaction was read at ELISA photometer at 490 nm and the absorbance of biotin-labelled cells after passage in the bottom compartment was measured. In some assays, PBMC (25 × 10^3^) from NHS or from MS patients at baseline were exposed with 100 nM FTY720 for 24 h and then placed in chemotaxis chamber for assay as above described. Chemotactic activity was assessed as *migration index* calculated according to the following formula^34^:

$${\rm{Absorbance}}\,{\rm{of}}\,{\rm{biotin}}-{\rm{labelled}}\,{\rm{cells}}/{\rm{absorbance}}\,{\rm{of}}\,{\rm{negative}}\,{\rm{control}}.$$

### Statistical analysis

Multiple comparisons were performed with ANOVA and Tukey post test for parametric data. Comparisons between paired groups was performed with Wilcoxon matched-pairs signed rank test for non-parametric data. Normality of data was estimated with D’Agostino & Pearson normality test. Correlation analysis was performed with Spearman’s test and Pearson r coefficient for non-parametric and parametric data, respectively. P < 0.05 values were considered significant. All statistical tests were performed with GraphPad Prism (version 7.02), San Diego, CA, USA.

## Electronic supplementary material

Supplementary Table S1, supplementary figure S1, S2 and S3

## Data Availability

All data generated or analysed during this study are included in this published article (and its Supplementary Information files).

## References

[1] Compston A, Coles A (2008). Multiple sclerosis. Lancet.

[2] Forster C (2008). Tight junctions and the modulation of barrier function in disease. Histochem. Cell Biol..

[3] Kazmierski R (2012). Serum tight-junction proteins predict hemorrhagic transformation in ischemic stroke patients. Neurology.

[4] Ingwersen J (2012). Fingolimod in multiple sclerosis: mechanisms of action and clinical efficacy. Clin. Immunol..

[5] Foster CA (2009). FTY720 rescue therapy in the dark agouti rat model of experimental autoimmune encephalomyelitis: expression of central nervous system genes and reversal of blood-brain-barrier damage. Brain Pathol..

[6] Nishihara H (2015). Fingolimod Prevents Blood-Brain Barrier disruption induced by the sera from patients with Multiple Sclerosis. PLoS One.

[7] Yanagida K (2017). Size-selective opening of the blood–brain barrier by targeting endothelial sphingosine 1–phosphate receptor 1. Pnas.

[8] Noseworthy JH (2000). Multiple sclerosis. N. Engl J. Med..

[9] Plumb J (2002). Abnormal endothelial tight junctions in active lesions and normal-appearing white matter in multiple sclerosis. Brain Pathol..

[10] Kirk J (2003). Tight junctional abnormality in multiple sclerosis white matter affects all calibres of vessel and is associated with blood–brain barrier leakage and active demyelination. J. Pathol..

[11] Claudio L, Raine CS, Brosnan CF (1995). Evidence of persistent blood–brain barrier abnormalities in chronic progressive multiple sclerosis. Acta Neuropathol..

[12] Minagar A (2003). Serum from patients with multiple sclerosis downregulates occludin and VE-cadherin expression in cultured endothelial cells. Mult. Scler..

[13] Smith ME (1993). Clinical worsening in multiple sclerosis is associated with increased frequency and area of gadopentate-dimeglumine- enhancing magnetic resonance imaging lesions. Ann. Neurol..

[14] Weibe S (1992). Serial cranial and spinal cord magnetic resonance imaging in multiple sclerosis. Ann. Neurol..

[15] Barkhof F (1992). Relapsing-remitting multiple sclerosis: sequential enhancing MR imaging vs clinical findings in determining disease activity. A.J.R. Am. J. Roentgenol..

[16] Cotton F (2003). MRI contrast uptake in new lesions in relapsing-remitting MS followed at weekly intervals. Neurology.

[17] McCaffrey G (2007). Tight junctions contain oligomeric protein assembly critical for maintaining blood–brain barrier integrity *in vivo*. J. Neurochem..

[18] Ballabh P, Braun A, Nedergaard M (2004). The blood–brain barrier: an overview. Structure, regulation, and clinical implications. Neurobiol. Dis..

[19] Lassmann H, van Horssen J, Mahad D (2012). Progressive multiple sclerosis: pathology and pathogenesis. Nat. Rev. Neurol..

[20] Pham TH, Okada T, Matloubian M, Lo CG, Cyster JG (2008). S1P1 receptor signaling overrides retention mediated by G alpha-coupled receptors to promote T cell egress. Immunity.

[21] Oo ML (2007). Immunosuppressive and anti-angiogenic sphingosine 1-phosphate receptor-1 agonists induce ubiquitinylation and proteasomal degradation of the receptor. J. Biol. Chem..

[22] Henault D (2013). Basis for fluctuations in lymphocyte counts in fingolimod-treated patients with multiple sclerosis. Neurology.

[23] Blumenfeld S, Staun-Ram E, Miller A (2016). Fingolimod therapy modulates circulating B cell composition, increases B regulatory subsets and production of IL-10 and TGFb in patients withMultiple Sclerosis. J. Autoimm..

[24] Thomas K (2017). Fingolimod additionally acts as immunomodulator focused on the innate immune system beyond its prominent effects on lymphocyte recirculation. J. Neuroinflamm..

[25] Novakova L (2017). Cerebrospinal fluid biomarkers of inflammation and degeneration as measures of fingolimod efficacy in multiple sclerosis. Mult. Scler. J..

[26] Zhao Y (2018). Fingolimod targets cerebral endothelial activation to block leukocyte recruitment in the central nervous system. J. Leuk. Biol..

[27] Waubant E (2006). Biomarkers indicative of blood-brain barrier disruption in multiple sclerosis. Dis. Markers.

[28] Hawkins CP, Mackenzie F, Tofts P, Du Boulay EP, McDonald WI (1991). Patterns of blood-brain barrier breakdown in inflammatory demyelination. Brain.

[29] McDonald WI (2001). Recommended diagnostic criteria for multiple sclerosis: guidelines from the International Panel on the diagnosis of multiple sclerosis. Ann. Neurol..

[30] AIFA. Gilenya©. Riassunto delle caratteristiche del prodotto. http://farmaci.agenziafarmaco.gov.it/aifa/10/06/2016 (2016).

[31] Kurtzke JF (1983). Rating neurologic impairment in multiple sclerosis: an expanded disability scale (EDSS). Neurology.

[32] De Santi L (2009). Higher expression of BDNF receptor gp145trkB is associated with lower apoptosis intensity in T cell lines in multiple sclerosis. J. Neurol. Sci..

[33] Pearce-Pratt R, Phillips DM, Bourinbaiar AS (1991). Simple colorimetric cell-cell adhesion assay using biotinylated lymphocytes. J. Immunol. Methods.

[34] Capitani N (2012). S1P1 expression is controlled by the pro-oxidant activity of p66Shc and is impaired in B-CLL patients with unfavorable prognosis. Blood.

