# Supplementary material for: Fingolimod reduces circulating tight-junction protein levels and in vitro peripheral blood mononuclear cells migration in multiple sclerosis patients
Source: Sci Rep. 2018 Oct 18;8:15371. doi: 10.1038/s41598-018-33672-9 (PMC6193926; doi:10.1038/s41598-018-33672-9)

Supplementary information for:

Fingolimod reduces circulating tight-junction protein levels and in vitro peripheral blood mononuclear cells migration in multiple sclerosis patients

PASQUALE ANNUNZIATA*, CHIARA CIONI, GIANNI MASI, MARISTELLA TASSI°, GIUSEPPE MAROTTA°, SAURO SEVERI°°

Department of Medicine, Surgery and Neurosciences, University of Siena, Italy

° Stem Cell Transplant and Cellular Therapy Unit, University Hospital, Siena, Italy

°° Neurology Unit, San Donato Hospital, Arezzo, Italy

***Corresponding author:**

Pasquale Annunziata, MD

Dipartimento di Scienze Mediche, Chirurgiche e Neuroscienze

Università di Siena

Unità di Neuroimmunologia clinica

Viale Bracci, 2

53100 SIENA, ITALY

Tel.: +39-0577-233482

Supplementary Table S1

Correlation between serum tight-junction protein levels and clinical and MRI features

	**RR at baseline**	**RR at T_{12}**	**EDSS at baseline**	**EDSS at T_{12}**	**New MRI T2-weighted lesions**	**MRI T1 GAD^{+} lesions**
**OCLN**	0.01	0.34	-0.25	-0.27	-0.25	-0.14
**CLN-5**	-0.02	0.18	-0.31	-0.39	0.12	-0.12
**ZO-1**	-004	-0.14	0.32	0.23	-0.05	-0.23

RR = relapse rate; T_{12} = at 12 months of therapy; EDSS = expanded disability status scale; GAD^{+} = gadolinium enhancing lesions; OCLN = occludin; CLN-5 = claudin-5; ZO-1 = zonula occludens -1. The results are expressed as r values. Spearman rank correlation was calculated. No r value is statistically significant.

Supplementary Figure S1: Representative ELISA standard curves of recombinant occludin (OCLN) **(A**), claudin-5 (CLN-5) (**B**) and zonula occludens-1 (ZO-1) (**C**). O.D. = optical density.

Supplementary Figure S2: Titration ELISA curves of recombinant occludin (OCLN) **(A**), claudin-5 (CLN-5) (**B**) and zonula occludens-1 (ZO-1) (**C**). O.D. = optical density. NHS = normal healthy subjects. MS = multiple sclerosis. The results are expressed as mean absorbance values (read at 490 nm wavelength) ±SEM of triplicate values of serum samples from 2 NHS and 2 MS patients. In all curves, background absorbance values obtained omitting serum samples were subtracted to the absorbance values of serum samples.

Supplementary Figure S3: Competitive ELISA of respective tight-junction proteins in the presence of increasing concentrations of occludin (OCLN) or bovine serum albumin (BSA) **(A)**, of claudin-5 (CLN-5) or BSA **(B)** and of zonula occludens-1 (ZO-1) or BSA **(C).** O.D. = optical density. Each point is the mean of triplicate values.

Supplementary Figure S1

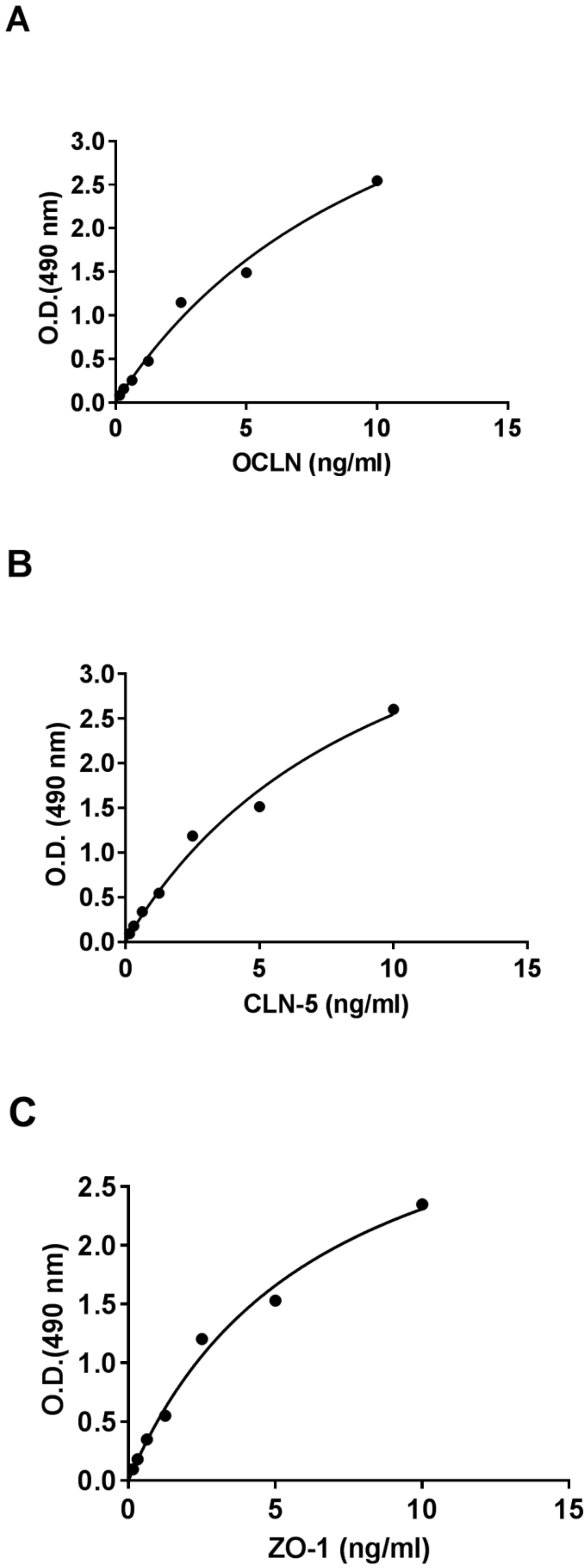

Supplementary Figure S2

A

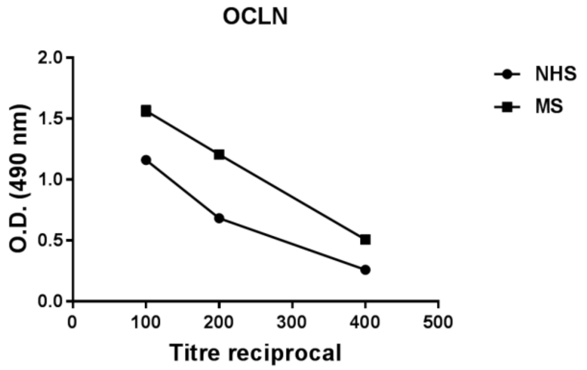

B

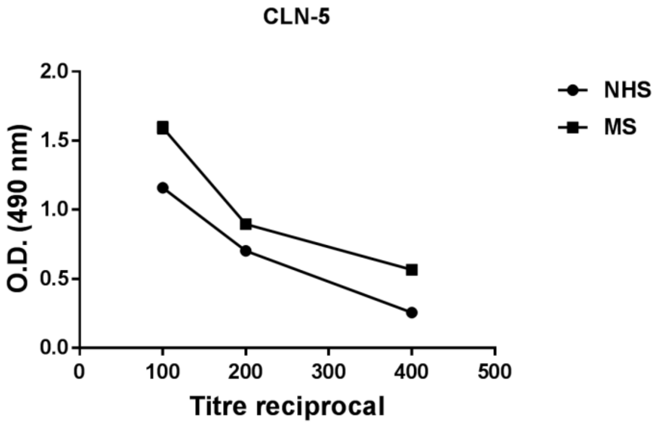

C

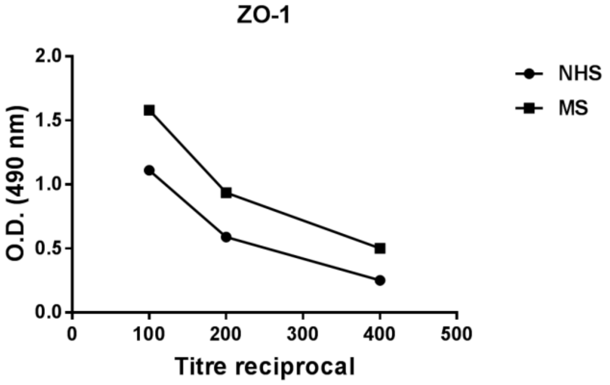

Supplementary Figure S3

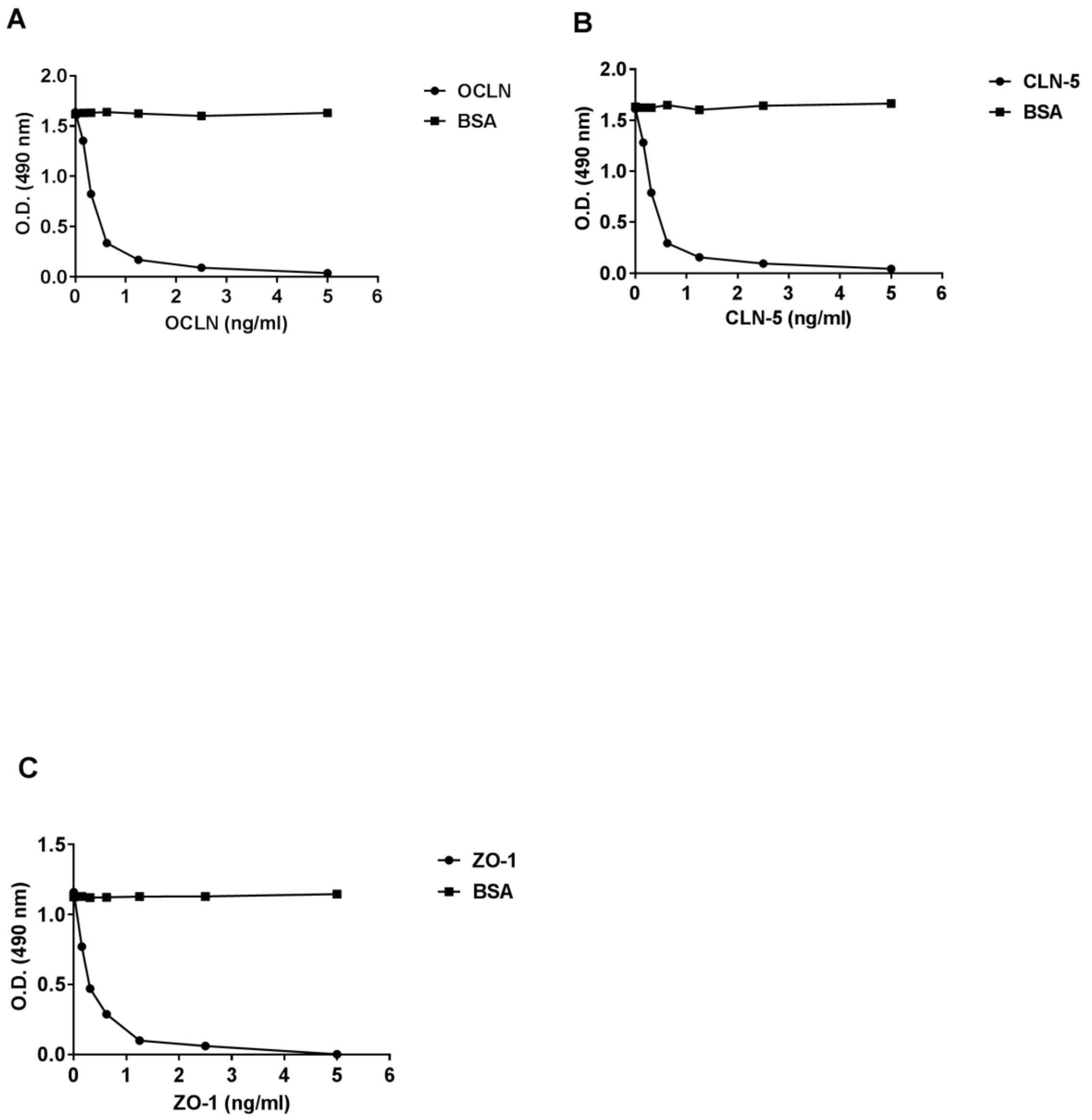

Supplement: Supplementary file 1 — Supplementary Table S1, supplementary figure S1, S2 and S3 [file 41598_2018_33672_MOESM1_ESM.pdf]
